# Efficacy of highly bioavailable oral curcumin in asymptomatic or mild COVID-19 patients: a double-blind, randomized, placebo-controlled trial

**DOI:** 10.1186/s41043-024-00584-6

**Published:** 2024-06-24

**Authors:** Atsuhiro Kishimoto, Maki Komiyama, Hiromichi Wada, Noriko Satoh-Asahara, Hajime Yamakage, Yoichi Ajiro, Hiroki Aoyama, Yasuhiro Katsuura, Atsushi Imaizumi, Tadashi Hashimoto, Yoichi Sunagawa, Tatsuya Morimoto, Masashi Kanai, Hideaki Kakeya, Koji Hasegawa

**Affiliations:** 1 Therabiopharma Inc., Kawasaki City, Kanagawa Japan; 2 Clinical Research Institute, NHO Kyoto Medical Center, Kyoto City, Kyoto Japan; 3 Department of Metabolic Syndrome and Nutritional Science, Research Institute of Environmental Medicine, Nagoya University, Nagoya City, Aichi Japan; 4 Niijuku Co-Op Clinic, Tokyo, Japan; 5 Division of Clinical Research, National Hospital Organization Yokohama Medical Center, Yokohama City, Kanagawa Japan; 6 Division of Molecular Medicine, School of Pharmaceutical Sciences, University of Shizuoka, Shizuoka City, Shizuoka Japan; 7 Graduate School of Medicine, Kyoto University, Kyoto City, Kyoto Japan; 8 Graduate School of Pharmaceutical Sciences, Kyoto University, Kyoto City, Kyoto Japan

**Keywords:** COVID-19, Curcumin, curcuRouge^®^, Clinical trial, Saturation of percutaneous oxygen, Body temperature

## Abstract

**Introduction:**

Even after the peak of the COVID-19 pandemic, the number of mild cases remains high, requiring continuous control. Curcumin, owing to its anti-inflammatory properties, can suppress vital proliferation and cytokine secretion in animal models. We developed a highly absorbable curcumin, curcuRouge^®^ (cR), which is approximately 100 times more orally bioavailable than conventional curcumin. We evaluated the effect of cR on the inhibition of disease progression in asymptomatic or mildly symptomatic COVID-19 patients.

**Methods:**

This study evaluated the effect of 7-day oral intake of cR (360 mg twice daily). Patients within 5 days of COVID-19 diagnosis were randomly assigned to a placebo or cR group in a double-blind manner.

**Results:**

Primary endpoint events [body temperature (BT) ≥ 37.5 °C and saturation of percutaneous oxygen (SpO2) < 96%] were fewer than expected, and the rate of these events was 2.8% in the cR group (2/71) and 6.0% in the placebo group (4/67); hazard ratio (HR) = 0.532, 95% confidence interval (CI) 0.097–2.902. Patients receiving cR tended to take fewer antipyretic medications than those receiving placebo (HR = 0.716, 95% CI 0.374–1.372). Among patients with a normal range of BT at baseline, the BT change rate was significantly (*p* = 0.014) lower in the cR group (− 0.34%) versus placebo (− 0.01%).

**Conclusion:**

The relative suppression of event rates and antipyretic medications taken, and significant decrease of subclinical BT support the anti-inflammatory effects of cR in asymptomatic or mildly symptomatic patients with COVID-19.

*Trial registration*: Japan Registry of Clinical Trials (CRB5200002).

**Supplementary Information:**

The online version contains supplementary material available at 10.1186/s41043-024-00584-6.

## Introduction

The COVID-19 pandemic had a significant global impact, causing not only health problems but also economic damage. Most COVID-19 patients are asymptomatic or mildly ill, but some develop hypoxemia (i.e., moderate disease), severe illness requiring ventilation (i.e., severe disease), and even succumb to multiple organ failure [1]. The risk factors for progression from asymptomatic or mild disease to moderate or severe disease include advanced age, male sex, hypertension, diabetes, cardiovascular disease, chronic kidney disease, chronic obstructive pulmonary disease, and obesity (body mass index > 30) [2]. Patients with mild or moderate disease or at greater risk for severe disease are eligible for hospitalization or antiviral medications. Unfortunately, antivirals are expensive and medical resources are limited. There is no recommended treatment for other asymptomatic or mildly ill patients, and they should be monitored at home or in residential care facilities. However, the number of asymptomatic and mildly ill patients remains extremely high [2], and there are frequent waves of infection, with the 8th and 9th waves peaking in January and August–September 2023 in Japan, respectively [2]. Although there were few severe cases in the 8th and 9th waves, the former had a record number of patients [2]. Currently, COVID-19 is seen in transmission cycles along with influenza, and thus it is necessary to continue countermeasures against asymptomatic and mildly ill patients.

Curcumin, which is used as a health food, exhibits anti-inflammatory effects by suppressing the activation of the transcription factor nuclear factor-kappa B (NF-κB). We have shown that curcumin inhibits NF-κB signaling by CRISPR-Cas9 screening [3]. In an influenza pneumonia animal model, curcumin has suppressed the expression of interleukin-6 and tumor necrosis factor-α in lung tissue [4]. Curcumin also has inhibitory effects on severe acute respiratory syndrome coronavirus 2 (SARS-CoV-2) 3CL protease or transmembrane protease, serine 2 (TMPRSS2) serine protease, which is required for viral growth and infection [5, 6]. Curcumin is insoluble in water and has extremely low bioavailability. We have developed a highly bioavailable curcumin formulation, curcuRouge^®^ (cR), which reaches a blood concentration approximately 100 times higher than that of general curcumin and the pharmacokinetics of cR was described in our previous report [7–9]. Furthermore, we found that cR strongly reduced the neutrophil/lymphocyte ratio, an important prognostic marker in COVID-19, in elderly patients [10]. The ratio of C-reactive protein (CRP) to lymphocytes (CLR) is known to be an important prognostic marker for COVID-19 [11–13], and cR has been shown to reduce serum C-reactive protein levels [14]. On the other hand, several COVID-19 clinical trials using curcumin preparations [15–17] have shown some improvement in symptoms, but most evaluated its combination with standard therapy or piperine, etc., rather than as a single agent such as in this study. Furthermore, most research focused on patients with moderate/severe disease, but the current trend shows more patients with asymptomatic or mild disease. Based on the current knowledge, curcumin may reduce the disease progression of asymptomatic and mild COVID-19 by inhibiting SARS-CoV-2 proliferation and inflammation. However, curcumin is difficult to give in a clinical setting because of its poor absorption rate when taken orally. Therefore, this study aimed to evaluate the efficacy of cR in preventing the progression of asymptomatic and mild COVID-19 to moderate disease.

## Materials and methods

### Validation in COVID-19 patient volunteers

The trial was approved by the Nara Medical University Ethics Committee and registered with the Japan Registry of Clinical Trials (CRB5200002). The primary endpoint was the rate of events, namely body temperature (BT) ≥ 37.5 °C, progression to SpO2 of < 96%, and hospitalization or nonhospitalized death up to 7 days after the start of test food. The study was conducted at the NHO Kyoto Medical Center (Kyoto, Japan) from February 2022 to January 2023. Patients who met all of the following criteria were included in the study: (1) diagnosis of COVID-19 within the past 5 days by gene amplification or antigen test (as described in the Guide to the Diagnosis and Treatment of Novel Coronavirus Infections (COVID-19), 10th Edition (Ministry of Health, Labor and Welfare of Japan), (2) SpO2 ≥ 96 on room air, (3) BT < 37.5 °C (4) nonhospitalized, (5) Eastern Cooperative Oncology Group Performance Status (ECOG PS) of 0 or 1, (6) males or females ≥ 20 years old, (7) provided written informed consent. The exclusion criteria were as follows: (1) taking antithrombotic drugs (e.g., antiplatelet agents, anticoagulants, ethyl icosapentate preparations, prostacyclin preparations), (2) with a history of cerebral hemorrhage, (3) on home oxygen therapy or dialysis, (4) with terminal cancer, (5) regular steroid use, (6) regular users of health foods containing curcumin, (7) having a history of allergy to curcumin, (8) pregnant and nursing women or those who might become pregnant, (9) patients for whom neutralizing antibody therapy is decided, and (10) other subjects who are deemed inappropriate by the principal investigator and sub-investigators for participation in this study. Informed consent was obtained in advance from all subjects participating in this study in the form of an explanatory consent document approved by the Ethical Review Board.

### Test food

This study used curcuRouge^®^ (cR) and placebo capsules, which were generated as previously described [7] and provided by Therabiopharma, Inc. Notably, cR is a highly absorbable curcumin formulation approximately 100 times greater bioavailability than regular curcumin, and each capsule contains 90 mg of curcumin. The placebo (control food) contained cornstarch instead of curcumin and was adjusted to have the same color tone as cR using food dye. Identical opaque capsules were used for both cR and the placebo, making it impossible to distinguish between the two from the outside.

### Assignment

Before the start of the study, the statistician assigned a test food number to cR and placebo using a random number table and sent this to the person responsible for allocation and Therabiopharma, Inc. Only the study statistician and the person responsible for allocation had information on the correspondence table between the test food number and the test food (cR and placebo). Others involved in the study (i.e., principal investigators and subinvestigators) were blinded to this information. Based on the allocation, Therabiopharma, Inc. marked the bottles filled with capsules of the test food with the test food number and sent them to the Clinical Research Office of Kyoto Medical Center; it was impossible to identify whether the capsules were cR or placebo. The clinical research office stored and managed the test food in an appropriate place where temperature and humidity could be controlled, avoiding direct sunlight. We recruited participating subjects by posting recruitment articles on the websites of medical institutions. Adjustment factors for assignment included age (< 60 years, > 60 years), gender (male, female), a number of risk factors [hypertension, diabetes, cardiovascular disease, chronic kidney disease, chronic obstructive pulmonary disease, obesity (BMI > 30)], and vaccination history.

### Dosing

curcuRouge^®^ (cR) capsules (90 mg/capsule as curcumin) or placebo capsules were taken orally, 4 capsules twice daily (morning and evening) for 7 days. cR used in this study is a commonly available health food. We have confirmed that there are no safety problems by a 28-day repeated-dose toxicity study in rats with 300 mg/kg/day (18,000 mg/day for a human weighing 60 kg, or 2900 mg/day at the human equivalent dose) (unpublished data). We have confirmed that a 30–180 day administration of cR 180 mg/day in humans is safe and effective by previous multiple clinical studies [10, 14]. This manuscript deals with a short-term, 7-day study to evaluate the effect of cR on the pathological condition of infection. Considering the risk–benefit, we set the dose at 720 mg/day, which is four times the dose of 180 mg/day that was found to be effective for the biomarkers related to inflammation and immunity and one-fourth the dose of 2900 mg/day that was confirmed safe.

### Combination therapy

Concomitant use of basic therapeutic agents (oral, inhalation, patch, application, or self-injection) due to preexisting medical history was allowed. Sputum regulators, antitussives, antibiotics, and nonsteroidal anti-inflammatory drugs [non-steroidal anti-inflammatory drug (NSAIDs)] were also allowed during the study period, depending on the symptoms. Patients were subsequently excluded from the study if the decision was made to administer anti-viral agents for COVID-19.

### Rationale for setting target number of cases

Because there are no reports on the effect of cR in COVID-19, we attempted to predict the event rate and establish the number of patients needed for the study. The study was planned for 2021, and reports from China at the time indicated that 81% and 19% of the overall COVID-19 population had asymptomatic/mild and moderate/severe disease [1]. A stratified analysis in a clinical trial of cocktail antibodies (Casilivimab and Imdevimab) reported that 42% of asymptomatic patients with confirmed infection taking placebo progressed to symptomatic mild illness [18]. Based on this data, we assumed a 42% rate of events (progression to SpO2: < 96, progression to BT ≥ 37.5 °C, and death during hospitalization or nonhospitalization) in the placebo group and a 21% rate of progression to moderate disease with cR (progression to moderate disease is 1/2 of the placebo group in the cR group). Assuming a dropout rate of about 14% during the follow-up period, 80 patients with asymptomatic/mild COVID-19 were needed in each of the placebo and cR groups, for a total of 160 cases.

### Statistical analyses

The Full Analysis Set was used as the analysis population, and analyses were performed using Fisher’s exact establishment test or t-test for unpaired samples to compare the two groups at baseline. Event-free survival was defined as the time from the start of treatment to the earliest date when an event of the primary endpoint was confirmed; this was evaluated using Kaplan–Meier curves. The effect of covariates on the composite event was analyzed using a Cox proportional hazards model with the allocation adjustment factor as a covariate. One-sided 5% alpha levels and two-sided 95% confidence intervals were used for testing. Cox proportional hazards analysis for the event (Andersen–Gill’s recurrent event model analysis) was used to determine the number of antipyretics taken. Corresponding t-tests and two-way ANOVA were used for BT, blood pressure, and SpO2. The Statistical Package for Social Sciences software program, version 24.0, for Windows (IBM Japan, Tokyo, Japan) was used for statistical analysis.

## Results

**Fig. 1:**
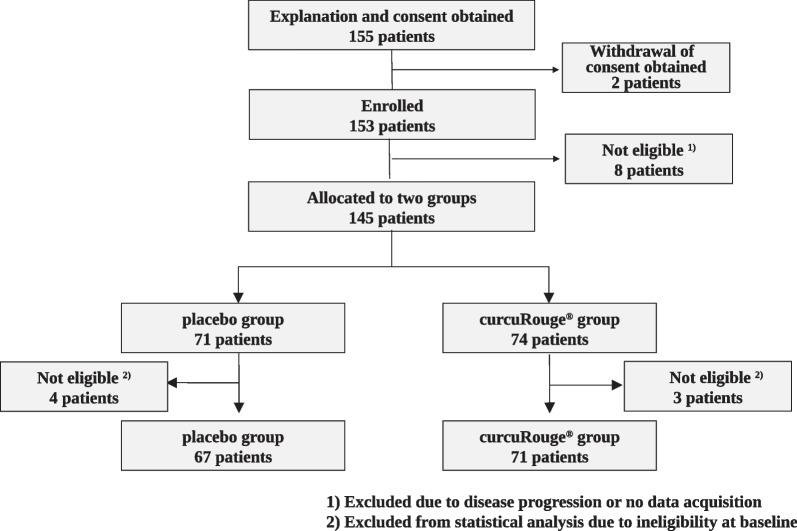
Study flow chart

A flowchart of the study population is shown in Fig. 1. Initially, informed consent was obtained from 155 patients, who had asymptomatic/mild COVID-19 and were within 5 days from the diagnosis of COVID-19 by gene amplification or antigen testing. Two patients withdrew consent before allocation. Then, 8 patients were excluded due to disease progression or to no data acquisition because of lost contact. Finally, 145 patients were randomly and dynamically assigned using allocation adjustment factors to placebo or cR group. After the observation period ended, seven patients (placebo, n = 4; cR, n = 3) were found to be ineligible and excluded from full analysis set because they had SpO2 < 96 or body temperature ≥ 37.5 °C at baseline (on Day 0). Background information (BT, SpO2, BMI, blood pressure, and pulse rate) on the day before starting treatment is shown in Table 1.

**Table 1:** Baseline characteristics

	Placebo		curcuRoge^®^		*P* value	
	n	Data	n	Data		
Number of patients	67		71			
Age	65	46.5 ± 13.1	70	48.0 ± 12.5	0.508	a
Gender	64		70		0.858	b
Women		41, 64.1		43, 61.4		
Men		23, 35.9		27, 38.6		
BMI	62	22.1 + 3.5	65	22.9 ± 3.3	0.245	a
Number of vaccines	65	2.8 ± 1.0	70	2.8 ± 1.0	> 0.999	a
Smoking history	61	5, 8.2	66	11, 16.7	0.186	b
*Personal history*						
High blood pressure	67	8, 11.9	71	7, 9.9	0.788	b
Diabetes	67	3, 4.5	71	1, 1.4	0.355	b
Cardiovascular disease	67	0, 0.0	71	2, 2.8	0.497	b
Chronic kidney disease	67	0, 0.0	71	0, 0.0		b
Chronic obstructive pulmonary disease	67	1, 1.5	71	3, 4.2	0.620	b
Cancer	67	3, 4.5	71	1, 1.4	0.355	b
Days from positive test to start of medication	60	3.7 ± 1.6	65	3.5 ± 1.5	0.455	a
Systolic blood pressure (SBP)	54	115.0 ± 16.2	55	118.8 ± 14.8	0.208	a
Diastolic blood pressure (DBP)	54	73.7 ± 8.9	55	75.2 ± 11.7	0.449	a
Stroke of pulse	0		1	54.0		a
Body temperature	56	36.1 + 0.4	56	36.3 ± 0.4	0.037	a
Sp02	57	98.0 + 1.0	56	97.9 ± 1.1	0.786	a

Data n, % or mean ± sd

*P* value a, unpaired t test b, Fisher's exact test

**Fig. 2:**
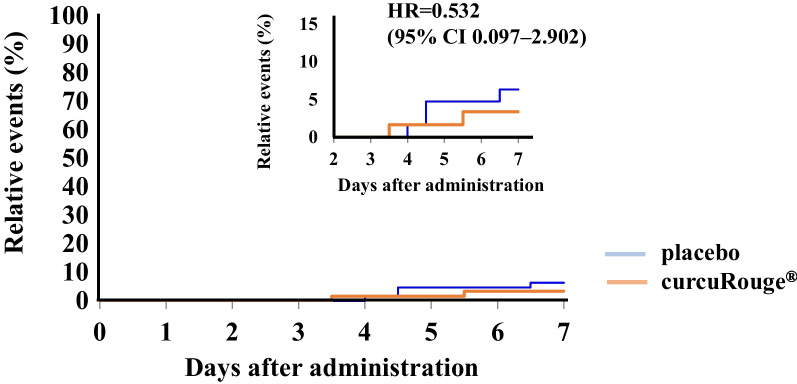
Kaplan–Meier curves of primary end points (SpO2 < 96 or body temperature ≥ 37.5℃) in placebo and curcuRouge^®^ groups

At baseline, there were no differences in age, gender distribution, number of vaccines, number of days from positive viral infection to intiation of medication, blood pressure, SpO2, smoking and alcohol history, and history of preexisting conditions. BT was significantly higher in the cR group (Table 1). Next, the effect of cR was tested with respect to the primary endpoint (i.e., the occurrence of events with SpO2 < 96 and BT ≥ 37.5 °C) (Fig. 2). Only events occurring on or after the third day of medication were included in the analysis, because early events during the first two days of the study were likely influenced by baseline background factors. There were no events of hospitalization or nonhospitalized death. Events (SpO2 < 96) occurred in 2 patients in the cR group (n = 1 on day 3; n = 1 on day 5) and in 4 patients in the placebo group (n = 3 on day 4; n = 1 on day 6). At the time of the study design, the event rate was estimated to be 42% because alpha and delta strains were the main infectious agents. However, almost all patients in this study were considered to be infected with omicron strains. The event rate in the placebo group was extremely low at 6.0%, whereas the rate was lower in the cR at 2.8% than the placebo (HR = 0.532, 95% CI 0.097–2.902) (Fig. 2).

There were 12 and 13 patients in the placebo and cR groups, respectively, who took NSAIDs during the study period, but the number of NASIDs taken per patient was lower in the cR group (2.2 ± 3.2) than in the placebo group (3.3 ± 3.2; *p* = 0.239). Andersen–Gill’s recurrent event model analysis, wherein NSAID compliance was considered an event, also showed lower events in the cR group than in the placebo group (HR = 0.716, 95% CI 0.374–1.372).

**Fig. 3:**
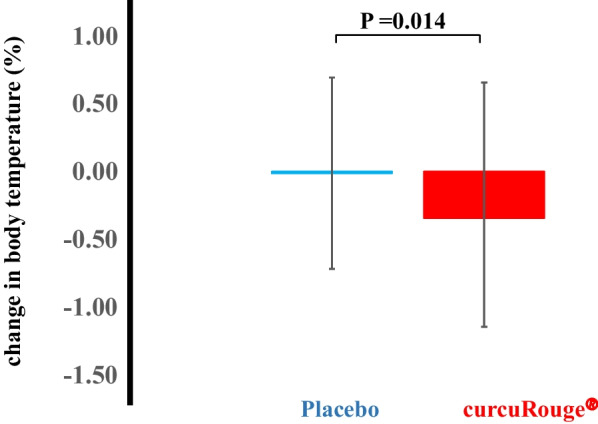
Percentage changes of body temperature from Day-1 to Day-7 in the placebo (n = 64) and curcuRouge^®^ (n = 66) groups

The effects of cR on subclinical conditions were then examined. This analysis included 64 and 66 patients in the placebo and cR groups, respectively, with a normal BT (≥ 35.5 °C and ≤ 37.0 °C) at baseline (before medication). Changes in BT, systolic blood pressure (SBP), diastolic blood pressure (DBP), and SpO2 were analyzed during the study period (Fig. 3, Supplementary Table S1). Comparing the start and end dates of treatment, there was a significant decrease in BT in the cR group (*p* = 0.001), but no change in the placebo group (*p* = 0.884). The decrease in BT over time was significantly greater in the cR group versus the placebo group (*p* for interaction = 0.014). SpO2 increased in both groups, both at a similar degree. There were no differences in SBP or DBP over time or between the groups. A similar trend was observed in patients who did not take antipyretics from the start date to the last day of medication (Supplementary Table S2).

## Discussion

Several clinical trials investigating the effects of curcumin on COVID-19 patients have been conducted [15–17], mainly in hospitalized patients with moderate or severe disease, with approximately 30 patients per group. Curcumin has been reported to improve symptoms such as SpO2 and respiration versus placebo. This study aimed to evaluate the effect of cR on the inhibition of progression of asymptomatic and mild COVID-19 to moderate disease. BT and SpO2, which are used to diagnose severe disease, were used as the main indices for this purpose. As shown in Fig. 2, there was a 47% reduction in event occurrence between curcuRouge^®^ (cR) and placebo (2.8% vs. 6.0%), but this was not statistically validated because the event rate was extremely low compared to that assumed at the time of study design. Although the study plan designed the number of cases based on the event rate of the alpha and delta strains, almost all patients in the study were considered to be infected with the omicron strain. Furthermore, vaccination was limited to two doses at the start of the study, but subsequently increased to a maximum of five doses by the end of the study. These factors led to an extremely low event rate of 6.0% in the placebo group in this study, and the rate to detect the effect of cR may have been lower than expected. When examining the number of cases required for a significant difference based on the event rate in the placebo group in this study and the event suppression effect of cR, 930 cases per group were required (α = 0.05 [two-tailed], power 0.8). Therefore, a larger study is needed to validate the effect of cR in reducing the incidence of events.

Because the baseline BT was higher in the cR group (Table 1), we conducted an analysis in patients with a normal BT on the day of initiation of treatment (Supplementary Table S1). These results suggest that cR may have a BT-lowering effect in COVID-19 patients (Fig. 3). The number of oral NASIDs taken was also lower in the cR group. In pathogen-induced fever, innate immune cells in the blood are able to sense pathogen-related molecules, such as viral proteins, and produce thermogenic intermediates (e.g., cytokines and prostaglandins) which act on the preoptic area to produce fever [19]. These activated immune cells produce reactive oxygen species and cause inflammation in the surrounding tissues, such as lung cells [20]. We recently demonstrated that oral administration of cR in patients with knee osteoarthritis significantly reduced serum C-reactive protein levels and the number of oral NASIDs, which are typical markers of inflammation, and significantly improved joint function and pain level (based on Japanese Orthopedic Association score and Visual Analog Scale score, respectively) [14]. The CLR is known to be an important prognostic marker for COVID-19 [11–13], and cR has the potential to lower CLR. Curcumin has also been shown to inhibit influenza growth in vitro [21], and the binding of SARS-CoV-2 to angiotensin converting enzyme-2 protein [22]. These findings suggest that the anti-inflammatory and antiviral effects of cR may be responsible for the decrease in BT in COVID19 patients.

## Conclusion

In this study, we examined the effect of cR, a highly absorbable curcumin, on preventing the progression of asymptomatic/mild COVID-19 to moderate disease. cR reduced event occurrence by 47%, but the number of events was so small that the results were not statistically significant. Nevertheless, cR tended to reduce event rates and the number of antipyretic medications taken, as well as significantly lowered BT in normothermic patients compared to placebo. These findings may reflect the subclinical effects of the drug, which should be clarified in larger studies.

### Supplementary Information

**Additional file 1: Table S1.** Change of parameters from Day-1 to Day-7 in curcuRouge^®^ and placebo groups.

**Additional file 2: Table S2.** Change of parameters from Day-1 to Day-7 in curcuRouge^®^ and placebo groups of patients who did not take antipyretics.

## Data Availability

The datasets used and/or analysed during the current study are available from the corresponding author on reasonable request.

## Abbreviations

COVID-19: Coronavirus disease-2019
SARS-CoV-2: Severe acute respiratory syndrome coronavirus 2
NF-κB: Nuclear factor-kappa B
TMPRSS2: Transmembrane protease, serine 2
SpO2: Saturation of percutaneous oxygen
SBP: Systolic blood pressure
DBP: Diastolic blood pressure
ECOG PS: Eastern Cooperative Oncology Group Performance Status
NSAID: Non-steroidal anti-inflammatory drug
BT: Body temperature
CRP: C-reactive protein
CLR: The ratio of CRP to lymphocytes
